# Variability of the Chemical Composition and Bioactivity between the Essential Oils Isolated from Male and Female Specimens of *Hedyosmum racemosum* (Ruiz & Pav.) G. Don

**DOI:** 10.3390/molecules26154613

**Published:** 2021-07-29

**Authors:** Eduardo Valarezo, Vladimir Morocho, Luis Cartuche, Fernanda Chamba-Granda, Magdaly Correa-Conza, Ximena Jaramillo-Fierro, Miguel Angel Meneses

**Affiliations:** Departamento de Química, Universidad Técnica Particular de Loja, Loja 110150, Ecuador; svmorocho@utpl.edu.ec (V.M.); lecartuche@utpl.edu.ec (L.C.); dalila1989.-@hotmail.com (F.C.-G.); juliscorream@hotmail.com (M.C.-C.); xvjaramillo@utpl.edu.ec (X.J.-F.); mameneses@utpl.edu.ec (M.A.M.)

**Keywords:** *Hedyosmum racemosum*, essential oils, chemical composition, α-phellandrene, *Klebsiella pneumoniae*

## Abstract

*Hedyosmum racemosum* (Ruiz & Pav.) G. is a native species of Ecuador used in traditional medicine for treatment of rheumatism, bronchitis, cold, cough, asthma, bone pain, and stomach pain. In this study, fresh *H. racemosum* leaves of male and female specimens were collected and subjected to hydrodistillation for the extraction of the essential oil. The chemical composition of male and female essential oil was determined by gas chromatography–gas chromatography equipped with a flame ionization detector and coupled to a mass spectrometer using a non-polar and a polar chromatographic column. The antibacterial activity was assayed against five Gram-positive and two Gram-negative bacteria, and two dermatophytes fungi. The scavenging radical properties of the essential oil were evaluated by DPPH and ABTS assays. The chemical analysis allowed us to identify forty-three compounds that represent more than 98% of the total composition. In the non-polar and polar column, α-phellandrene was the principal constituent in male (28.24 and 25.90%) and female (26.47 and 23.90%) essential oil. Other main compounds were methyl chavicol, germacrene D, methyl eugenol, and α-pinene. Female essential oil presented a strong activity against *Klebsiella pneumoniae* (ATCC 9997) with an minimum inhibitory concentration (MIC) of 500 μg/mL and a scavenging capacity SC_50_ of 800 µg/mL.

## 1. Introduction

The Chloranthaceae family consists of herbs, shrubs, or small aromatic trees and includes the genera *Hedyosmum* (45 accepted species), *Chloranthus* (14 accepted species), *Ascarina* (12 accepted species), and *Sarcandra* (2 accepted species) [1]. This family, considered one of the most primitive among the angiosperms, is characterized by the presence of secretory cells in the stems and leaves [2].

In America, *Hedyosmum* is the most abundant genus in the Chlorantaceae family [3]. The species of this genus are widespread in low and high mountain rain forests at altitudes of approximately 500 to 3000 m above sea level [4]. They are commonly found in the mountains of southern Mexico, Central America, and the Andes of South America (Brazil, Ecuador, Peru, and central Bolivia) [5,6,7].

**Figure 1:**
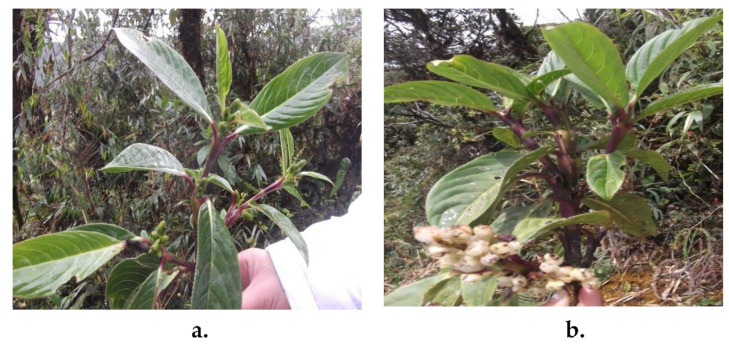
Specimens of *Hedyosmum racemosum*: (**a**) male and (**b**) female.

The genus *Hedyosmum* can be found in the form of trees or shrubs, with branches bulging or hinged at the height of the nodes. Its leaves are simple, opposite, toothed, and petiolate with pinnate veins [8]. When the foliage is crushed, it becomes strongly aromatic; indeed, the genus name finds its origin in the Greek *hedy* (pleasant) and *osmum* (smell), referring to this smell that is associated with pepper, lemon, and anise [9]. The species of the genus *Hedyosmum* are dioecious, and some have male and female inflorescences. The male inflorescences are arranged in the form of terminal or axial spikes, while the female inflorescences are arranged in the form of racemes or panicles (Figure 1). Individuals with male inflorescences are usually called male specimens or simply “male”; in the same way, female specimens are called “female”. The fruits are small, fleshy, and white when ripe [9]. Only the female flowers produce fruits. Figure 1 shows the tender white fruits of the female specimen (end of the flowering stage and beginning of the fruiting stage).

Regarding the chemical composition, the genus *Hedyosmum* presents as major components sesquiterpenes, sesterterpenes, derivatives of hydroxycinnamic acid, flavonoids, neolignans, and others. The genus *Hedyosmum* is also an important source of essential oils (EOs) that can be extracted from different parts of plants, such as aerial parts, leaves, flowers, and fruits. The chemical composition and the potential for different applications of these EOs have been described in numerous studies [6,7,10].

The species of the genus *Hedyosmum* also present various traditional uses, for example, as sources of firewood and construction materials, as well as sources of food (drinks and fruits) and medicinal preparations, mainly in the form of an infusion of leaves [8]. The main pharmacological effects associated with members of this genus are neuroprotective, anxiolytic, antidepressant, sedative, analgesic, antinociceptive, anticancer, antibacterial, and anti-plasmodial effects [11].

The leaves of different Hedyosmum species have been used to make aromatic beverages, including *H. uniflorum*, *H. anisodorum*, *H. cumbalense*, *H. strigosum*, *H. scaberrimum*, and *H. purpurascens*. On the other hand, the species *H. anisodorum*, *H. arborescens*, *H. scaberrimus*, *H. sprucei*, and *H. scabrum* have been reported as digestives, antispasmodics, and stomach calming (anxious or nervous stomach), while *H. bonplandianum*, *H. angustifolim*, and *H. racemosum* are used to soothe rheumatic joint pain as well as fever and cold symptoms. The traditional use of *H. cuatrecazanum* and *H. luteynii* is associated with the treatment of kidney problems. *H. goudotianum* is traditionally used for headaches, and *H. sprucei* has been used in the treatment of snake bites [9,12].

In Ecuador, which is one of the countries with the greatest biodiversity in the world and where ancestral traditions are still practiced [13], 16 species of the genus *Hedyosmum* are reported [11]. Among these species, *Hedyosmum racemosum* (Ruiz & Pav.) G. Don is a native species widely used in traditional Ecuadorian medicine, as an antibacterial and for the treatment of rheumatism, bronchitis, cold, cough, asthma, bone pain, stomach pain, and the nervous system [13]. In Ecuador, *H. racemosum* is known as “Alcopa”, “Asarcito”, “Masamoche”, “Asarquiro”, “Choleta”, “Acha guayusa”, “Úntuntup” (Shuar chicham), “Guayusa de monte”, and “Jicamilla grande” (Castilian). This species is found to be distributed in different Ecuadorian provinces, such as Cañar, Carchi, Loja, Morona-Santiago, Napo, Pastaza, Pichincha, Sucumbíos, and Zamora [12].

Despite the fact that *H. racemosum* has extensive ancestral knowledge, in the scientific literature, there is not enough information on the chemical composition and biological activity of the volatile compounds of this species. For this reason, this study presents for the first time the comparison of the essential oils of male and female specimens of the *H. racemosum* species, being an important starting point for future research in the field of phytochemistry and pharmacology.

## 2. Results

### 2.1. Isolation and Physical Properties of Essential Oil

The essential oil was isolated from fresh leaves of male and female Hedyosmum racemosum specimens. The moisture and yield in essential oil of the leaves, and physical properties of the essential oil obtained, are shown in Table 1. The essential oil in both cases was a translucent viscous liquid, and some samples showed a slightly yellowish hue.

### 2.2. Essential Oil Compounds’ Identification

The identification of volatile compounds presents in EO of the male and female *Hedyosmum racemosum specimens* was carried out by GC/MS and GC/FID. The qualitative and quantitative data of the chemical composition obtained using nonpolar column DB-5 ms are shown in Table 2. Forty-one compounds were identified in the essential oil isolated from male specimens (male EO) and forty-three in the essential oil isolated from female specimens (female EO); in both cases, a percentage greater than 99% of the total composition was identified.

**Figure 2:**
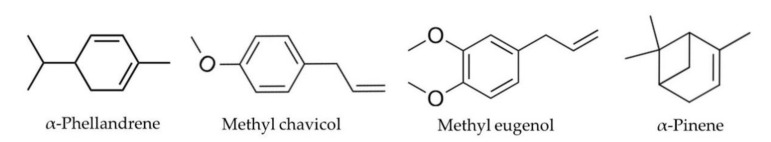
Main compounds of the essential oil from male and female specimens of *Hedyosmum racemosum*.

**Figure 3:**
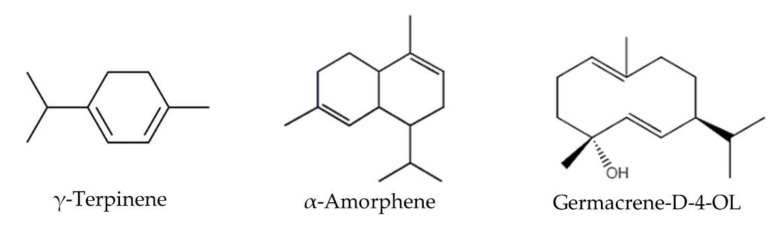
Compounds identified only in female *Hedyosmum racemosum* essential oil.

The oil compounds of the male and female specimens identified using nonpolar column were mainly grouped into aliphatic monoterpene (ALM) hydrocarbons with a representation of 48.39% and 46.75%, respectively. In addition, the compounds grouped as oxygenated monoterpenes (OXMs) represented 28.93% in male EO and 24.51% in female EO. Compounds belonging to the aromatic sesquiterpene hydrocarbons (ARS) group were not identified. The ALM α-phellandrene (compound 7, CF: C_10_H_16_, MM: 136.13 Da) was the principal constituent in male (28.24 ± 1.39) and female (26.47 ± 2.95) essential oil. Other main compounds (>5%) were methyl chavicol (21), germacrene D (34), methyl eugenol (30), and α-pinene (2) (Figure 2). Compounds 10 and 11 (limonene and β-phellandrene), eluted together (co-eluted), both represented 3.68% in male EO and 3.36% in female EO. Small amounts of γ-terpinene (15) and α-amorphene (35) (Figure 3) were identified only in female EO.

The data of the chemical composition qualitative and quantitative of the male EO and female EO obtained using polar column HP-INNOWax are shown in Table 3. Forty-one compounds were identified in male EO, which represent 98.83% of the total composition, and forty-three compounds in female EO (99.62%).

The compounds of the male EO and female EO identified using the polar column were mainly grouped into ALM with a representation of 45.82% and 43.63%, respectively. The principal compound was α-phellandrene, with 25.90 ± 1.97% in male EO and 23.90 ± 1.89% in female EO. Other main compounds were 21, 2, 34, and 30. As for the non-polar column γ-terpinene, a minority compound (<5%) was identified only in female EO. In addition, the compound germacrene-D-4-OL (Figure 3) was identified in female EO. Compounds belonging to the ARS group were not identified.

Essential oils of leaves from male and female specimens of *H. racemosum* were assessed by the microdilution broth method againt Gram-negative bacteria, Gram-positive bacteria, and dermatophytes fungi, and the values of minimum inhibitory concentration (MIC, μg/mL) are shown in Table 4. At the maximum evaluated concentration of 1000 µg/mL, the female EO reported an MIC of 500 µg/mL against *Klebsiella pneumoniae* (ATCC 9997).

**Figure 4:**
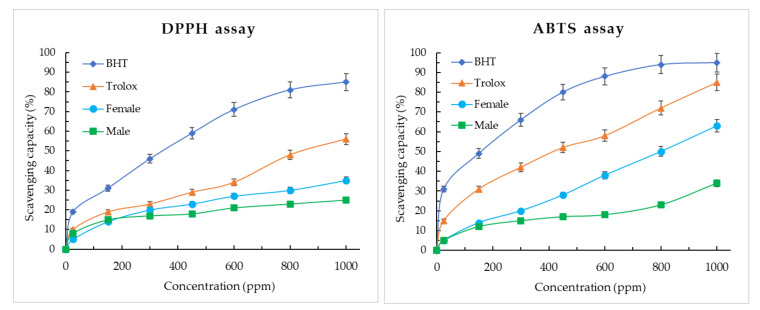
Antioxidant activity of *Hedyosmum racemosum* leaves essential oil.

The male and female EO of *H. racemosum* was explored for antioxidant activity using DPPH^•^ and ABTS^•+^ scavenging activity. BTH and Trolox were used as positive control and the results obtained are shown in Figure 4.

SC_50_ was used as a measure of the value of inhibition concentration of 50% of the activity (Table 5). Through the DPPH method, male and female essential oils showed little antioxidant activity with 25% and 35% of inhibition to 1000 ppm, but did not provide SC_50_ values at the maximum concentration tested (1000 μg/mL). Among standards tested, BHT (SC_50_ = 350 ± 20 μg/mL) was the most efficient. Employing the ABTS technique, female EO showed an SC_50_ = 800 ± 30 μg/mL, male EO did not reach SC_50_ values in the concentration ranges tested, while BHT reported an SC_50_ = 170 ± 10 μg/mL.

## 3. Discussion

The yield of the essential oil extracted from male specimens was near similar to that obtained from female specimens. The chemical profile of male and female EO *H. racemosum* specimens was shown to be similar qualitatively, both in the polar column and non-polar column; however, they have light quantitatively differences. This chemical composition was different from other *Hedyosmum* species. Unlike the data in this study, where the principal groups of oil compounds were similar for male and female specimens, Herrera et al. [11] in 2018 reported a different relation for *H. scabrum* having oxygenated monoterpenes and monoterpene hydrocarbons as the principal groups of female EO, while for male EO, the predominant groups were sesquiterpenes and oxygenated monoterpenes.

Considering the main chemical components of EO *Hedyosmum* species summarized by Radice et al. [9], the EO of *H. racemosum* in coincidence with other *Hedyosmum* species presents four main components: α-pinene (*H. angustifolium* and *H. scabrum* male and female specimen), α-phellandrene (*H. arborescens*, *H. Brasiliense*, and *H. sprucei*), linalool (*H. Angustifolium* and *H. scabrum*), and germacrene D (*H. scabrum*, *H. sprucei*, *H traslucidum*, and *H. costaricence*). Similarly, the EO *H. racemosum* presents sabinene and β-pinene, which are common compounds in the EO of *Hedyosmum* species. Torres et al. [14] in 2018 reported the chemical composition of EO of *Hedyosmum luteynii* Todzia from the main compounds; α-phellandrene (32.72%), α-pinene (13.2%), and germacrene D (3.2%) were also identified in EO *H. racemosum* as main compounds.

The three main chemical components of EO from *H. racemosum* were α-phellandrene, methyl chavicol (synonym estragole), and germacrene D, while for the EO of *H. scabrum* male and female specimens [11], these compounds were non-detected; however, other minor components were reported in the EO of male and female, but in different proportions: α-pinene (15% and 6.4%), linalool (16.5% and 3.3%), and methyl eugenol (1% and 0.6%). The chemical similarity of the EO of these *Hedyosum* species was 39.46% and 41.67% for male and female EO, respectively. Likewise, in the EO of female *H. racemosum* specimens, three components were identified that are not present in male EO: γ-terpinene (0.06%), germacrene-D-4-ol (0.19%), and α-amorphene (0.14%). It can be considered that the absence of chemotypes, because the majority compound (α-phellandrene), was the same in both oils.

According to the results of inhibitory activity (MIC >1000 μg/mL), the EO of *H. recemosum* does not present good activity against gram-positive pathogenic bacteria and fungi species tested; regarding the gram-negative bacterias, the female EO specie *H. racemosum* show an MIC of 500 μg/mL against *Klebsiella pneumoniae* (ATCC 9997). For a classification of the biological activity of EO, Van Vuuren and Holl in 2017 suggest that MIC values between 101 to 500 μg/mL can be reported as strong activity [15]. For all cases, the positive control shows an MIC lower than 4 μg/mL for bacterial species and equal to 20 μg/mL for fungi. The strong activity against *K. pneumoniae* indicates that the female EO of *H. recemosum* can be used to combat neumonal infections; moreover, this result is important when resistance of this bacteria to antiobiotics has been recognized. Kichner et al. in 2010 reported appreciable antibacterial activity of *Hedyosmum brasiliense* EO against *S. aureus*, *S. saprophyticus*, and *B. subtilis* with an MIC value of 0.312% (*v*/*v*) [3].

The essential oils are a complex mixture of compounds and the antimicrobial activity depends on the active principle and the synergism and antagonisms among compounds. Hence, taking into account the difference in the activity against *K. pneumonia* of the EO of male and female species of *H. racemosum*, it can be assumed that the strong antibacterial activity of the female EO of *H. racemosum* could be associated with the difference of the percentages of compounds; for example, 1.8-cineole, (Z)-β-ocimene, methyl chavicol, and germacrene D, and to compounds present only in female EO: γ-terpinene, germacrene-D-4-OL, and α-amorphene. γ-Terpinene, a monoterpene, has been reported to have diverse biological activities as an antioxidant for protecting methyl linoleate, DNA, and erythrocyte [16], and as an antibacterial against rice pathogens [17].

The antioxidant results for DPPH and ABTS assays indicate a weak scavenging capacity; at the concentrations evaluated, only the female EO of *H. racemosum* allows to calculate the SC_50_ at a value of 800 μg/mL for ABTS assay. Murakami et al. in 2017 reported the antioxidant activity for male EO and female EO species of *H. brasiliense* for flowers with an SC_50_ of 2516.18 μg/mL and 3162.79 μg/mL, respectively; meanwhile, for leaves, they found an SC_50_ of 3783.49 μg/mL and 3542.01 μg/mL, respectively [10]. Anthony et al. [18] analyzed the antioxidant activity of pure essential oil components and concluded that hydrocarbons present very low antioxidant activity, but phenol compounds are more active followed by mono- and sesquiterpenes; from these data, it can be assumed that the antioxidant activity of EO depends on the abundance of more active compounds and explains the lower or no antioxidant activity for some EO.

Although some of the main compounds identified in the of *H. racemosum* EO (α-pinene, α-phellandrene, and germacrene D) are not considered as effective antioxidants [18], methyl chavicol (estragole), an oxygenated monoterpene, reported an SC_50_ of 312.5 μg/mL for DPPH assay [19]. Essential oils from *Ocimum basilicum* and *Tagetes lucida*, containing methyl chavicol as the main compound (17.06% and 95.7%, respectively), reported an SC_50_ of 1.09 μg/mL [20] and 37.9 μg/mL [21], respectively, indicating that the antioxidant activity in EOs is related to the synergistic and antagonistic action among the components. In addition, Gogoi et al. [22] reported the DPPH SC_50_ of pure methyl eugenol of 2.253 μg/mL and 2.263 μg/mL of a methyl eugenol-rich (73.17%) essential oil. As in the case of the antibacterial activity of female EO, the ABTS SC_50_ can be related to the antioxidant activity of the minor compounds γ-Terpinene [16].

## 4. Materials and Methods

### 4.1. Materials

Sodium sulfate anhydrous, dichloromethane (DCM), methanol (MeOH), 6-hydroxy-2,5,7,8-tetramethylchroman-2-carboxylic acid (Tolox), butylated hydroxytoluene (BHT), 2,2-diphenyl-1-picrylhydryl (DPPH), and 2,2′-azinobis-3-ethylbenzothiazoline-6-sulfonic acid (ABTS) were purchased from Sigma-Aldrich (San Luis, MO, USA). Dimethylsulfoxide (DMSO) was obtained from Fisher (Hampton, NH, USA). Microbiological media as Sabourad and Mueller Himton Broth were purchased from DIPCO (Quito, Ecuador). Aliphatic hydrocarbons standard was purchased from CHEM SERVICE (West Chester, PA, USA). Helium was purchased from INDURA (Quito, Ecuador). All chemicals were of analytical grade and used without further purifications.

### 4.2. Plant Material

The collection was performed with the authorization of the Ministerio del Ambiente de Ecuador (MAE) N° 001-IC-FLO-DBAP-VS-DRLZCH-MA. The leaves of the male and female specimens were collected in the “El Tiro” sector, located between the provinces of Loja and Zamora Chinchipe, at a latitude of 3°59′26″ S and a longitude of 79°08′45″ W. The collection was performed at an altitude of 2760 m a.s.l. The leaves of the two specimens were collected at the end of the flowering season and the beginning of the fruiting season. The storage and transfer of the plant material were performed using airtight plastic containers. The collection and transfer temperature was 16–18 °C (room temperature) and the pressure was around 89 KPa (room pressure). The botanical identification of specimens was performed at the herbarium of the “Universidad Nacional de Loja” by Dr. Bolivar Merino. A voucher specimen is preserved in the Herbarium of the Universidad Técnica Particular de Loja.

### 4.3. Postharvest Treatments

The postharvest treatments were done immediately after vegetal material arriving at the laboratory, between 1 and 2 h after being collected, and consist of the separation of foreign material and degraded leaves.

### 4.4. Moisture Determination

The moisture of plant material was determined using test method AOAC 930.04-1930, loss on drying (doisture) in plants. For moisture determination, an analytical balance (Mettler AC 100, Columbus, OH, USA) was determined.

### 4.5. Essential Oil Extraction

For the extraction of the essential oil, the vegetal material was hydrodistillated for 4 h in a clevenger-type apparatus, for which the sample mixed with water was boiled to evaporate volatile components, and then two layers (aqueous- and oil-rich) were obtained and oil was separated via a separating funnel. Subsequently, the moisture in the collected essential oil was removed by the addition of anhydrous sodium sulphate and, finally, it was stored in amber sealed vials at 4 °C to protect it from light until being used in the subsequent analysis composition [23].

### 4.6. Determination of Physical Properties of Essential Oil

The density was determined using the standard AFNOR NF T 75-111 [24] (equivalent to the standard ISO 279:1998) and, for the refractive index, the standard AFNOR NF T 75-112 [24] (similar to ISO 279:1998) was used. The density was performed using an analytical balance (model Mettler AC 100, ±0.0001) and a pycnometer (1 mL) and, for the refraction index, a refractometer (model ABBE) was used. Measurements were performed at 20 °C.

### 4.7. Identification of Chemical Constituents of the Essential Oil

#### 4.7.1. Quantitative Analysis

For quantitative analysis, an Agilent gas chromatograph (GC) (model 6890N series, Agilent Technologies, Santa Clara, CA, USA) equipped with a flame ionization detector (FID) was used. The GC-FID analyses were performed using a nonpolar Agilent J&W DB-5 ms Ultra Inert GC column (30 m, 0.25 mm, 0.25 µm) (5%-phenyl-methylpolyxilosane), a polar Agilent J&W HP-INNOWax GC column (30 m, 0.25 mm, 0.25 µm) (polyethylene glycol), and an automatic injector (Agilent 7683 automatic liquid sampler, Agilent Technologies, Santa Clara, CA, USA) in split mode. The samples, 1 µL of solution (1/100, *v*/*v*, EO/DCM), were injected with a split ratio of 1:50. Helium was used as a carrier gas at 1 mL/min in constant flow mode and at an average velocity of 25 cm/s. The initial oven temperature was held at 50 °C for 3 min and then it was heated to 230 °C with a ramp of 3 °C/min, and the temperature was maintained for 3 min until the end. The injector and detector temperatures were 250 °C. Quantification was done by external standard method using calibration curves generated by running GC analysis of representative compounds.

#### 4.7.2. Qualitative Analysis

For qualitative analysis, an Agilent gas chromatograph (model 6890N series, Agilent Technologies, Santa Clara, CA, USA) coupled to a mass spectrometer (quadrupole) detector (MS) (model Agilent series 5973 inert, Agilent Technologies, Santa Clara, CA, USA) was used. The GC-MS analyses were performed using the same columns and injector as GC-FID. The samples were injected with a split ratio of 1:50. Helium was used as a carrier gas at 0.9 mL/min in constant flow mode and at an average velocity of 34 cm/s. The operating conditions for the MS were as follows: electron multiplier 1670 eV, 70 eV, mass range 45–350 *m*/*z*, and scan rate 2 scan/s. MS was provided with a computerized system MSD-Chemstation D.01.00 SP1. The identification of the oil components was based on a comparison of mass spectrum data with the Wiley 7n libraries from the internal chromatograph database, and mass spectrum data and relative retention indices (RIs) with those of published literature [25,26,27]. RI of the compounds was determined based on the homologous of the standard aliphatic hydrocarbons, which were injected after the oils under the same conditions. The RI was obtained through the arithmetic index described by van Den Dool and Dec. Kratz [28] using Equation (1).

(1) $$\mathrm{RI}=100n+\frac{\left(\mathrm{RTx}-\mathrm{RTn}\right)}{\left(\mathrm{RTN}-\mathrm{RTn}\right)}$$

where n is the carbon number of the hydrocarbon that elutes before the compound of interest, RTx is the retention time (RT) of the compound of interest, RTn is the RT of the hydrocarbon that elutes before the compound of interest, and RTN is the RT of the hydrocarbon that elutes after the compound of interest.

### 4.8. Evaluation of Antimicrobial Activity

#### 4.8.1. Antibacterial Activity

The antibacterial activity of male and female essential oil was determined against five Gram-negative bacteria and two Gram-positive bacteria (Table 3) according to the procedure described by Valarezo et al. [29]. The bacterial strains were incubated in Müeller–Hinton (MH) broth and DMSO was used to dissolve the essential oil. Gentamicin was used as a positive control for all bacteria. DMSO was used as a negative control and the results are reported as minimum inhibitory concentration (MIC) (the lowest concentration of sample capable of inhibiting all visible signs of growth of the microorganism).

#### 4.8.2. Antifungal Activity

The antifungal activity of the essential oils was determined against two fungal organisms (Table 3) by the microdilution method according to the procedure described previously by Valarezo et al. [29]. Terbinafine was used as a positive control and DMSO was used as a negative control. The results are reported as minimum inhibitory concentration (MIC).

### 4.9. Antioxidant Capacity

#### 4.9.1. DPPH Radical Scavenging Activity

The 2,2-diphenyl-1-picrylhydryl radical (DPPH^•^) free radical scavenging activity of male and female essential oils was measured based on the technique of Brand Williams et al. [30] with some modifications described by Thaipong et al. [31]. The concentrations of *H. racemosum* essential oil used were 25, 150, 300, 450, 600, 800, and 100 ppm [29]. The samples were evaluated at a wavelength of 515 nm in a UV spectrophotometer (Genesys 10S UV.Vis Spectrophotometer, Thermo Scientific, Waltham, MA, USA). BHT and Trolox were used as a positive control and methanol as a blank control. Scavenging capacity (SC) was expressed as percentage and was calculated according to Equation (2).

(2) $$\mathrm{SC}\left(\%\right)=\frac{\left(\mathrm{As}-\mathrm{Ai}\right)}{\mathrm{As}}\times 100$$

where Ai is the absorbance of DPPH^•^ mixed with EO and As is the sample blank absorbance of DPPH^•^ in which EO has been replaced with methanol. The plotting of Sc (%) against the concentration of the essential oil allowed us to identify the oil concentration that provided 50% scavenging effect (SC_50_) of DPPH^•^.

#### 4.9.2. ABTS Radical Cation Scavenging Activity

The ABTS assay was performed according to the procedure described by Arnao et al. [32] with some modifications by Thaipong et al. [31] using 2,2′-azinobis-3-ethylbenzothiazoline-6-sulfonic acid radical cation (ABTS^•+^). The concentrations of *H. racemosum* essential oil used were 25, 150, 300, 450, 600, 800, and 100 ppm The samples were evaluated at a wavelength of 734 nm in a UV spectrophotometer (Genesys 10S UV.Vis Spectrophotometer, Thermo Scientific, Waltham, MA, USA). Like the DPPH analysis, BHT and Trolox were used as positive controls, while deionized water was used as blank control. Scavenging capacity (SC) was expressed as percentage and was calculated according to Equation (3).

(3) $$\mathrm{Sc}\left(\%\right)=\frac{\left(\mathrm{Ao}-\mathrm{Ai}\right)}{\mathrm{Ao}}\times 100$$

where Ao is the absorbance of ABTS^•+^ mixed with the sample and Ai is the absorbance after reaction of ABTS^•+^ with the sample. The plotting of Sc (%) against the concentration of the essential oil allowed us to identify the oil concentration that provided 50% scavenging effect (SC_50_) of ABTS^•+^.

### 4.10. Statistical Analysis

The procedures of essential oil extraction, determination of physical properties, identification of chemical constituents, and determination antioxidant capacity were repeated three times. The evaluation of antimicrobial activity was repeated nine times. Data were collected in Microsoft Excel, while the measures of central tendency and analysis of variance (ANOVA) were calculated using Minitab 17 (Version 17.1.0., Minitab LLC., State College, PA, USA). All results are expressed as mean values.

## 5. Conclusions

The chemical composition; physical properties; and antibacterial, antifungal, and antioxidant activities of essential oil from *Hedyosmum racemosum* leaves of specimens with male and female inflorescences were determined. The results show that all major compounds and most minor compounds present in male essential oil are the same as those determined in female essential oil; only two out of thirty-eight minority compounds were different between male and female essential oil. The percentages of the compounds were different between the oil of the male and female individuals, but in most cases, it was a non-significant statistical difference. The essential oil from specimens with female inflorescences showed more promising biological property values than the oil from specimens with male inflorescences. This study of chemical composition and bioactivity of male and female essential oil contributes to our knowledge concerning the variability of chemical compounds and biological properties between essential oils of male and female species. Furthermore, this study intends to motivate detailed investigations of the essential oils of other dioecious plant species.

## Figures and Tables

**Table 1:** Moisture and yield of the leaves and physical properties of the essential oil (EO).

	Male EO		Female EO	
Moisture (%)	61 ^a^	1 ^b^	58	1
Yield (%, *v*/*w*)	0.30	0.05	0.24	0.03
Density (g/cm^3^)	0.8978	0.0056	0.9038	0.0039
Refractive index, *n*^20^	1.4911	0.0015	1.4883	0.0018

^a^ Mean of nine determinations; ^b^ standard deviation.

**Table 2:** Chemical compounds present in the essential oil of *Hedyosmum racemosum* leaves identified using the non-polar column DB-5 ms.

CN	Compounds	RI	RI ^ref^	Male EO ^1^		Female EO ^2^		Type	CF	MM (Da)
				%	SD	%	SD			
1	α-Thujene	924	924	0.16	0.01	0.15	0.01	ALM	C_10_H_16_	136.13
2	α-Pinene	932	932	7.48	1.62	5.43	1.13	ALM	C_10_H_16_	136.13
3	Camphene	947	946	0.77	0.11	1.08	0.60	ALM	C_10_H_17_	137.13
4	Sabinene	968	969	0.98	0.15	0.92	0.29	ALM	C_10_H_16_	136.13
5	β-Pinene	973	974	2.14	0.40	1.74	0.43	ALM	C_10_H_16_	136.13
6	Myrcene	988	988	0.59	0.05	0.54	0.05	ALM	C_10_H_16_	136.13
7	α-Phellandrene	1003	1002	28.24	1.39	26.47	2.95	ALM	C_10_H_16_	136.13
8	δ-3-Carene	1011	1008	0.11	0.01	0.10	0.03	ALM	C_10_H_16_	136.13
9	p-Cymene	1021	1020	1.35	0.18	1.15	0.23	ARM	C_10_H_14_	134.1
10, 11	Limonene + β-Phellandrene ^3^	1024	1024	3.68	0.19	3.36	0.28	ALM	C_10_H_16_	136.13
12	1.8-Cineole	1026	1026	1.65	0.13	2.26	0.36	OXM	C_10_H_18_O	154.14
13	(Z)-β-Ocimene	1034	1032	2.88	0.55	4.25	0.77	ALM	C_10_H_16_	136.13
14	(E)-β-Ocimene	1043	1044	0.66	0.28	1.01	0.25	ALM	C_10_H_16_	136.13
15	γ-Terpinene	1054	1054	-	-	0.06	0.03	ALM	C_10_H_16_	136.13
16	Terpinolene	1083	1086	0.73	0.04	1.65	1.26	ALM	C_10_H_16_	136.13
17	Linalool	1099	1095	4.19	1.26	3.51	0.97	OXM	C_10_H_18_O	154.14
18	Camphor	1141	1141	0.54	0.15	0.64	0.25	OXM	C_10_H_16_O	152.12
19	Terpinen-4-ol	1172	1174	0.26	0.12	0.07	0.05	OXM	C_10_H_18_O	154.14
20	α-Terpineol	1189	1186	0.33	0.05	0.19	0.07	OXM	C_10_H_18_O	154.13
21	Methyl chavicol	1196	1195	21.82	2.85	17.64	2.23	OXM	C_10_H_12_O	148.09
22	Citronellol	1225	1223	0.13	0.09	0.21	0.01	OXM	C_10_H_20_O	156.15
23	Linalyl acetate	1250	1254	0.21	0.03	0.24	0.06	OTC	C_12_H_20_O_2_	196.14
24	Bornyl acetate	1280	1284	1.00	0.47	1.76	0.16	OTC	C_12_H_20_O_2_	196.14
25	α-Terpinyl acetate	1343	1346	1.66	0.39	1.25	0.08	OTC	C_12_H_20_O_2_	196.14
26	Citronellyl acetate	1350	1350	0.11	0.02	0.16	0.05	OTC	C_12_H_22_O_2_	198.16
27	α-Copaene	1372	1374	0.89	0.26	1.60	0.73	ALS	C_15_H_24_	204.19
28	β-Cubebene	1382	1387	0.14	0.07	0.24	0.09	ALS	C_15_H_24_	204.19
29	β-Elemene	1385	1389	0.47	0.16	0.72	0.25	ALS	C_15_H_24_	204.19
30	Methyl eugenol	1402	1403	5.38	0.76	5.99	0.51	OTC	C_11_H_14_O_2_	178.10
31	trans-Caryophyllene	1415	1417	0.68	0.05	0.99	0.40	ALS	C_15_H_24_	204.19
32	α-Humulene	1449	1452	0.17	0.04	0.28	0.11	ALS	C_15_H_24_	204.19
33	γ-Muurolene	1476	1478	0.13	0.04	0.14	0.03	ALS	C_15_H_24_	204.19
34	Germacrene D	1478	1480	7.62	2.10	10.34	1.36	ALS	C_15_H_24_	204.19
35	α-Amorphene ^4^	1486	1483	-	-	0.14	0.03	ALS	C_15_H_24_	204.19
36	Bicyclogermacrene	1497	1500	0.65	0.21	0.69	0.09	ALS	C_15_H_24_	204.19
37	α-Muurolene	1501	1500	0.14	0.05	0.15	0.03	ALS	C_15_H_24_	204.19
38	Germacrene A	1507	1508	0.19	0.08	0.31	0.12	ALS	C_15_H_24_	204.19
39	γ-Cadinene	1513	1513	0.15	0.08	0.22	0.06	ALS	C_15_H_24_	204.19
40	δ-Cadinene	1521	1522	0.86	0.24	1.24	0.39	ALS	C_15_H_24_	204.19
42	α-Muurolol (=Torreyol)	1640	1644	0.20	0.09	0.35	0.08	OXS	C_15_H_26_O	222.20
43	β-Eudesmol	1645	1649	0.28	0.18	0.51	0.17	OXS	C_15_H_26_O	222.20
*Aliphatic monoterpene hydrocarbons (ALM)*				48.39		46.75				
*Aromatic monoterpene hydrocarbons (ARM)*				1.35		1.15				
*Oxygenated monoterpenes (OXM)*				28.93		24.51				
*Aliphatic sesquiterpene hydrocarbons (ALS)*				12.08		17.05				
*Oxygenated sesquiterpene (OXS)*				0.48		0.86				
Other compounds (OTC)				8.36		9.39				
Total identified				99.60		99.72				

CN: compound number; RI: calculated retention indices; RI^ref^: references retention indices; SD: standard deviation; CF: chemical formula; MM: monoisotopic mass; -: not detected. ^1^ Essential oil isolated from male specimens; ^2^ essential oil isolated from male specimens; ^3^ co-eluted compounds.^4^ Compound identified only in the non-polar column.

**Table 3:** Chemical compounds present in the essential oil of *Hedyosmum racemosum* leaves identified using the polar column HP-INNOWax.

CN	Compounds	RI	RI ^ref^	Male EO		Female EO		Type	CF	MM (Da)
				%	SD	%	SD			
1	α-Thujene	1038	1035	0.18	0.02	0.17	0.02	ALM	C_10_H_16_	136.13
2	α-Pinene	1035	1032	7.03	1.31	5.03	1.04	ALM	C_10_H_16_	136.13
3	Camphene	1066	1066	0.74	0.08	1.02	0.55	ALM	C_10_H_17_	137.13
4	Sabinene	1124	1126	1.00	0.13	0.93	0.29	ALM	C_10_H_16_	136.13
5	β-Pinene	1114	1118	2.04	0.34	1.65	0.41	ALM	C_10_H_16_	136.13
6	Myrcene	1160	1160	0.52	0.16	0.39	0.17	ALM	C_10_H_16_	136.13
7	α-Phellandrene	1158	1165	25.90	1.97	23.90	1.89	ALM	C_10_H_16_	136.13
8	δ-3-Carene	1141	1148	0.27	0.03	0.58	0.43	ALM	C_10_H_16_	136.13
9	p-Cymene	1278	1280	1.41	0.19	1.23	0.25	ARM	C_10_H_14_	134.1
10	Limonene	1194	1202	2.28	0.11	2.07	0.13	ALM	C_10_H_16_	136.13
11	β-Phellandrene	1204	1210	1.31	0.11	0.78	0.05	ALM	C_10_H_16_	136.13
12	1.8-Cineole	1212	1213	1.35	0.11	2.31	0.87	OXM	C_10_H_18_O	154.14
13	(Z)-β-Ocimene	1235	1232	3.00	0.52	4.31	0.73	ALM	C_10_H_16_	136.13
14	(E)-β-Ocimene	1251	1254	0.79	0.27	1.14	0.26	ALM	C_10_H_16_	136.13
15	γ-Terpinene	1242	1244	-	-	0.06	0.02	ALM	C_10_H_16_	136.13
16	Terpinolene	1287	1290	0.75	0.07	1.59	1.12	ALM	C_10_H_16_	136.13
17	Linalool	1559	1553	4.55	1.25	4.18	1.13	OXM	C_10_H_18_O	154.14
18	Camphor	1523	1532	0.52	0.13	0.62	0.25	OXM	C_10_H_16_O	152.12
19	Terpinen-4-ol	1606	1605	0.12	0.01	0.17	0.07	OXM	C_10_H_18_O	154.14
20	α-Terpineol	1704	1706	0.48	0.14	0.31	0.11	OXM	C_10_H_18_O	154.13
21	Methyl chavicol	1675	1675	23.50	3.14	19.03	7.33	OXM	C_10_H_12_O	148.09
22	Citronellol	1778	1772	0.57	0.09	0.64	0.24	OXM	C_10_H_20_O	156.15
23	Linalyl acetate	1562	1565	0.25	0.03	0.19	0.11	OTC	C_12_H_20_O_2_	196.14
24	Bornyl acetate	1578	1579	0.92	0.42	1.67	1.09	OTC	C_12_H_20_O_2_	196.14
25	α-Terpinyl acetate	1702	1706	1.68	0.39	1.35	0.09	OTC	C_12_H_20_O_2_	196.14
26	Citronellyl acetate	1656	1654	0.20	0.05	0.30	0.07	OTC	C_12_H_22_O_2_	198.16
27	α-Copaene	1493	1497	0.84	0.20	1.52	0.63	ALS	C_15_H_24_	204.19
28	β-Cubebene	1542	1544	0.12	0.05	0.23	0.06	ALS	C_15_H_24_	204.19
29	β-Elemene	1585	1586	0.48	0.14	0.76	0.19	ALS	C_15_H_24_	204.19
30	Methyl Eugenol	2025	2030	6.30	1.10	7.37	3.55	OTC	C_11_H_14_O_2_	178.10
31	trans-Caryophyllene	1587	1592	0.26	0.06	0.39	0.21	ALS	C_15_H_24_	204.19
32	α-Humulene	1659	1660	0.15	0.03	0.28	0.08	ALS	C_15_H_24_	204.19
33	γ-Muurolene	1682	1684	0.16	0.06	0.18	0.01	ALS	C_15_H_24_	204.19
34	Germacrene D	1700	1704	6.65	1.75	9.46	2.23	ALS	C_15_H_24_	204.19
36	Bicyclogermacrene	1725	1727	0.54	0.17	0.58	0.05	ALS	C_15_H_24_	204.19
37	α-Muurolene	1718	1716	0.15	0.02	0.11	0.04	ALS	C_15_H_24_	204.19
38	Germacrene A	2081	2078	0.25	0.10	0.36	0.05	ALS	C_15_H_24_	204.19
39	γ-Cadinene	2235	2228	0.15	0.04	0.32	0.03	ALS	C_15_H_24_	204.19
40	δ-Cadinene	1752	1748	1.08	0.29	1.59	0.41	ALS	C_15_H_24_	204.19
41	Germacrene-D-4-OL^1^	2061	2069	-	-	0.19	0.12	OXS	C_15_H_26_O	222.20
42	α-Muurolol (=Torreyol)	2203	2209	tr	-	0.08	0.02	OXS	C_15_H_26_O	222.20
43	β-Eudesmol	2250	2257	0.34	0.14	0.58	0.15	OXS	C_15_H_26_O	222.20
*Aliphatic monoterpene hydrocarbons (ALM)*				45.82		43.63				
*Aromatic monoterpene hydrocarbons (ARM)*				1.41		1.23				
*Oxygenated monoterpenes (OXM)*				31.10		27.25				
*Aliphatic sesquiterpene hydrocarbons (ALS)*				10.81		15.78				
*Oxygenated sesquiterpene (OXS)*				0.34		0.85				
Other compounds (OTC)				9.36		10.88				
Total identified				98.83		99.62				

tr: traces (<0.5). ^1^ Compound identified only in the polar column.

**Table 4:** Antimicrobial activity of essential oil from *Hedyosmum racemosum*.

Microorganism	Male EO (µg/mL)	Female EO (µg/mL)	Positive Control ^a^ (µg/mL)
Gram-negative bacteria			
*Escherichia coli* (ATCC 25922)	>1000	>1000	1.95
*Klebsiella pneumoniae* (ATCC 9997)	>1000	500	1.95
*Proteus vulgaris *(ATCC 8427)	>1000	>1000	1.95
*Pseudomonas aeruginosa * (ATCC 27853)	>1000	>1000	1.95
*Salmonella typhimurium* (LT2)	>1000	>1000	3.91
Gram-positive bacteria			
*Enterococcus faecalis * (ATCC 29212)	>1000	>1000	3.91
*Staphylococcus aureus * (ATCC 25923)	>1000	>1000	1.95
Dermatophytes Fungi			
*Trichophyton rubrum * (ATCC 28188)	>1000	>1000	20
*Trichophyton mentagrophytes* (ATCC 28185)	>1000	>1000	20

^a^ Gentamicin for all bacteria and Terbinafine for fungi.

**Table 5:** Antioxidant activity of essential oils of *H. racemosum*.

Sample	DPPH	ABTS
	SC_50_ * (μg/mL)	
Male EO	>1000	>1000
Female EO	>1000	800 ± 30
BHT	350 ± 20	170 ± 10
Trolox	840 ± 30	420 ± 20

* SC_50_ = scavenging capacity of 50%.

## Data Availability

Data is available from the authors upon reasonable request.

## References

[1] Cao C.-M., Peng Y., Shi Q.-W., Xiao P.-G. (2008). Chemical Constituents and Bioactivities of Plants of Chloranthaceae. Chem. Biodivers..

[2] Eklund H., Doyle J.A., Herendeen P.S. (2004). Morphological Phylogenetic Analysis of Living and Fossil Chloranthaceae. Int. J. Plant. Sci..

[3] Kirchner K., Wisniewski Jr A., Cruz A.B., Biavatti M.W., Netz D.J.A. (2010). Chemical composition and antimicrobial activity of *Hedyosmum brasiliense* Miq., Chloranthaceae, essential oil. Rev. Bras. Farmacogn..

[4] Bercion S., Martin M.-A., Baltaze J.-P., Bourgeois P. (2005). A new α-methylene γ-lactone sesquiterpene from *Hedyosmum arborescens*. Fitoterapia.

[5] Sandoya V., Saura-Mas S., Granzow-de la Cerda I., Arellano G., Macía M.J., Tello J.S., Lloret F. (2021). Contribution of species abundance and frequency to aboveground forest biomass along an Andean elevation gradient. For. Ecol. Manag..

[6] Zhang M., Liu D., Fan G., Wang R., Lu X., Gu Y.-C., Shi Q.-W. (2016). Constituents from Chloranthaceae plants and their biological activities. Heterocycl. Commun..

[7] Guerrini A., Sacchetti G., Grandini A., Spagnoletti A., Asanza M., Scalvenzi L. (2016). Cytotoxic Effect and TLC Bioautography-Guided Approach to Detect Health Properties of *Amazonian Hedyosmum* sprucei Essential Oil. Evid. Based Complement. Altern. Med..

[8] Todzia C.A., Keating R.C. (1991). Leaf Architecture of the Chloranthaceae. Ann. Mo. Bot. Gard..

[9] Radice M., Tasambay A., Pérez A., Diéguez-Santana K., Sacchetti G., Buso P., Buzzi R., Vertuani S., Manfredini S., Baldisserotto A. (2019). Ethnopharmacology, phytochemistry and pharmacology of the genus Hedyosmum (Chlorantaceae): A review. J. Ethnopharmacol..

[10] Murakami C., Cordeiro I., Scotti M.T., Moreno P.R.H., Young M.C.M. (2017). Chemical Composition, Antifungal and Antioxidant Activities of *Hedyosmum brasiliense* Mart. ex Miq. (Chloranthaceae) Essential Oils. Medicines.

[11] Herrera C., Morocho V., Vidari G., Bicchi C., Gilardoni G. (2018). Phytochemical Investigation of Male and Female *Hedyosmum scabrum* (Ruiz & Pav.) Solms Leaves from Ecuador. Chem. Biodivers..

[12] Torre L.d.l., Navarrete H., Muriel M.P., Macía Barco M.J., Balslev H. (2008). Enciclopedia de las Plantas Útiles del Ecuador.

[13] Tene V., Malagón O., Finzi P.V., Vidari G., Armijos C., Zaragoza T. (2007). An ethnobotanical survey of medicinal plants used in Loja and Zamora-Chinchipe, Ecuador. J. Ethnopharmacol..

[14] Torres Rodríguez S.H., Tovar Torres M.C., García V.J., Lucena M.E., Araujo Baptista L. (2018). Composición química del aceite esencial de las hojas de *Hedyosmum luteynii* Todzia (Chloranthaceae). Rev. Peru. Biol..

[15] Van Vuuren S., Holl D. (2017). Antimicrobial natural product research: A review from a South African perspective for the years 2009–2016. J. Ethnopharmacol..

[16] Li G.-X., Liu Z.-Q. (2009). Unusual Antioxidant Behavior of α- and γ-Terpinene in Protecting Methyl Linoleate, DNA, and Erythrocyte. J. Agric. Food Chem..

[17] Yoshitomi K., Taniguchi S., Tanaka K., Uji Y., Akimitsu K., Gomi K. (2016). Rice terpene synthase 24 (OsTPS24) encodes a jasmonate-responsive monoterpene synthase that produces an antibacterial γ-terpinene against rice pathogen. J. Plant. Physiol..

[18] Anthony K.P., Deolu-Sobogun S.A., Saleh M.A. (2012). Comprehensive Assessment of Antioxidant Activity of Essential Oils. J. Food Sci..

[19] Santos B.C.S., Pires A.S., Yamamoto C.H., Couri M.R.C., Taranto A.G., Alves M.S., Araújo A.L.d.S.d.M., de Sousa O.V. (2018). Methyl Chavicol and Its Synthetic Analogue as Possible Antioxidant and Antilipase Agents Based on the In Vitro and In Silico Assays. Oxidative Med. Cell. Longev..

[20] Li H., Ge Y., Luo Z., Zhou Y., Zhang X., Zhang J., Fu Q. (2017). Evaluation of the chemical composition, antioxidant and anti-inflammatory activities of distillate and residue fractions of sweet basil essential oil. J. Food Sci. Technol..

[21] Olivero-Verbel J., González-Cervera T., Güette-Fernandez J., Jaramillo-Colorado B., Stashenko E. (2010). Chemical composition and antioxidant activity of essential oils isolated from Colombian plants. Rev. Bras. De Farmacogn..

[22] Gogoi R., Loying R., Sarma N., Begum T., Pandey S.K., Lal M. (2020). Comparative Analysis of In-Vitro Biological Activities of Methyl Eugenol Rich *Cymbopogon khasianus* Hack., Leaf Essential Oil with Pure Methyl Eugenol Compound. Curr. Pharm. Biotechnol..

[23] Valarezo E., Ojeda-Riascos S., Cartuche L., Andrade-González N., González-Sánchez I., Meneses M.A. (2020). Extraction and Study of the Essential Oil of Copal (Dacryodes peruviana), an Amazonian Fruit with the Highest Yield Worldwide. Plants.

[24] Association française de normalisation (AFNOR) (2000). Huiles Essentielles. Tome 1, Échantillonnage et Méthodes D’analyse.

[25] Adams R.P. (2007). Identification of Essential Oil Components by Gas. Chromatography/Mass Spectrometry.

[26] NIST 05 (2005). Mass Spectral Library (NIST/EPA/NIH).

[27] NIST Libro del Web de Química del NIST, SRD 69. In Base de Datos de Referencia Estándar del NIST Número 69. http://webbook.nist.gov.

[28] van Den Dool H., Kratz P.D. (1963). A generalization of the retention index system including linear temperature programmed gas-liquid partition chromatography. J. Chromatogr. A.

[29] Valarezo E., Merino G., Cruz-Erazo C., Cartuche L. (2020). Bioactivity evaluation of the native Amazonian species of Ecuador: *Piper lineatum* Ruiz & Pav. essential oil. Nat. Volatiles Essent. Oils.

[30] Brand-Williams W., Cuvelier M.E., Berset C. (1995). Use of a free radical method to evaluate antioxidant activity. LWT Food Sci. Technol..

[31] Thaipong K., Boonprakob U., Crosby K., Cisneros-Zevallos L., Hawkins Byrne D. (2006). Comparison of ABTS, DPPH, FRAP, and ORAC assays for estimating antioxidant activity from guava fruit extracts. J. Food Compos. Anal..

[32] Arnao M.B., Cano A., Acosta M. (2001). The hydrophilic and lipophilic contribution to total antioxidant activity. Food Chem..

